# Gene-Based Nonparametric Testing of Interactions Using Distance Correlation Coefficient in Case-Control Association Studies

**DOI:** 10.3390/genes9120608

**Published:** 2018-12-05

**Authors:** Yingjie Guo, Chenxi Wu, Maozu Guo, Xiaoyan Liu, Alon Keinan

**Affiliations:** 1 School of Computer Science and Technology, Harbin Institute of Technology, Harbin 150001, China; liuxiaoyan@hit.edu.cn; 2 Department of Biological Statistics and Computational Biology, Cornell University, Ithaca, NY 14853, USA; alon.keinan@cornell.edu; 3 Department of Mathematics, Rutgers University, Piscataway, NJ 08854, USA; wuchenxi2013@gmail.com; 4 Beijing Key Laboratory of Intelligent Processing for Building Big Data, Beijing University of Civil Engineering and Architecture, Beijing 100044, China

**Keywords:** genome-wide association studies, qualitative trait, gene–gene interaction, distance correlation coefficient

## Abstract

Among the various statistical methods for identifying gene–gene interactions in qualitative genome-wide association studies (GWAS), gene-based methods have recently grown in popularity because they confer advantages in both statistical power and biological interpretability. However, most of these methods make strong assumptions about the form of the relationship between traits and single-nucleotide polymorphisms, which result in limited statistical power. In this paper, we propose a gene-based method based on the distance correlation coefficient called gene-based gene-gene interaction via distance correlation coefficient (GBDcor). The distance correlation (dCor) is a measurement of the dependency between two random vectors with arbitrary, and not necessarily equal, dimensions. We used the difference in dCor in case and control datasets as an indicator of gene–gene interaction, which was based on the assumption that the joint distribution of two genes in case subjects and in control subjects should not be significantly different if the two genes do not interact. We designed a permutation-based statistical test to evaluate the difference between dCor in cases and controls for a pair of genes, and we provided the *p*-value for the statistic to represent the significance of the interaction between the two genes. In experiments with both simulated and real-world data, our method outperformed previous approaches in detecting interactions accurately.

## 1. Introduction

Genome-wide association studies (GWAS) are a well-established and effective method of identifying genetic loci associated with common diseases or traits, and they have identified over 65,000 unique single-nucleotide polymorphisms (SNPs) that are associated with various traits or diseases [1,2,3,4,5]. Earlier GWAS analysis strategies were based largely on single-locus models, which tested the association between individual markers and a given phenotype independently. Although this type of approach has identified many regions of disease susceptibility successfully, most of the SNPs that have been identified have small effect sizes that failed to account fully for the heritability of complex traits. Genetic interaction has been hypothesized to play an important role in the genetic basis of complex diseases and traits, [6,7], and it has been one of the possible solutions to the problem of “missing heritability” [8,9,10]. Even if genetic interaction explains only a tiny fraction of “missing heritability”, it can still provide some biological insight into the pathway by aiding the construction of novel gene pathway topologies.

The first investigations on genetic interactions were at the SNP level, in which various statistical methods, which included logistic regression [11,12,13], odds-ratio [14], linkage disequilibrium (LD), [15,16,17], and entropy-based statistics [18,19], were employed to detect SNP–SNP interactions (i.e., epistasis). Other techniques that have been used to study SNP–SNP interactions include multifactor dimensionality reduction (MDR) [20], Tuned RelieF (TuRF) [21], Bayesian epistasis association mapping (BEAM) [6], Tree-based epistasis association mapping (TEAM) [22], Boolean operation-based screening and testing (BOOST) [23], and permutation-based Random Forest (pRF) [24]. These marker-based methods have encountered some common challenges, such as the complexity that arises from the large number of pairwise or higher-order tests because all pairs or groups of SNPs have to be considered and because of the extensive multiple testing correction, which weakens their statistical power. In this paper, we aim to improve the power of the detection of gene–gene interactions by moving beyond the SNP level and, instead, consider all potential pairs of SNPs from each of a pair of genes in a single, gene-based, interaction detection.

In the study of the main (marginal) associations in GWAS, gene-based approaches have been successful, and therefore, it might be worth extending it to the analysis of interaction between genes. There are several potential advantages of this approach. First, it can reduce the number of pairwise tests substantially, because there are usually many fewer genes than SNPs. For example, detection of pairwise, gene-based interactions for 20,000 genes requires ∼$2\times 10^{8}$ tests, but for three million SNPs, the marker-based interaction tests require more than ∼$5\times 10^{12}$. Second, gene-based approaches might have greater statistical power, because a gene contains more information than a single SNP and because there might be multiple ways for genes to interact with each other that are aggregated; this is also true when doing a gene-based study for main effects [25,26]. Third, it might be easier to incorporate prior biological knowledge with this approach (e.g., information on protein–protein interactions (PPI) or known membership of genes in pathways). Finally, the results of gene-based analysis may have more meaningful biological implications and be more interpretable.

In the work of Peng et al. [27], canonical correlation between two genes was performed on both the case and the control groups. A U-statistic called canonical correlation-based U statistic (CCU) was used to measure the difference in the correlation between these two genes, which was used to indicate the presence of interaction. A limitation of this method was that in the correlation analysis, only linear relations were considered. To overcome this limitation, Yuan et al. [28] and Larson et al. [29] extended CCU to kernelized CCU (KCCU), where the canonical correlation analysis was kernelized to account for possible non-linearity. Li et al. [30] introduced another method called the gene-based information gain method (GBIGM), which was entropy-based and non-parametric. More recently, Emily [31] developed a new method called gene-based gene-gene interaction test(AGGrEGATOr), which combined the *p*-values in marker-level interaction tests to measure the interaction between two genes. Earlier, this strategy was used successfully by Ma et al. [32] for the detection of interaction for quantitative phenotypes.

In this paper, we propose a novel method to identify gene-level, gene–gene interactions among case control studies of complex phenotypes based on the distance correlation coefficient called gene-based gene-gene interaction via distance correlation coefficient (GBDcor). Distance correlation [33,34,35] quantifies all types of dependent relationships between two random vectors with arbitrary, but not necessarily equal, dimensions, which is better than Pearson’s correlation, which only focuses on the linear relationship. Distance correlation has already been used in bioinformatics to detect co-expression genes [36] and imaging genetics associations [37]. We use the difference in dependence relationships between case samples and control samples as an indicator of gene–gene interaction, which is based on the assumption that the joint distribution of two genes in case subjects and in control subjects should not be significantly different if the two genes do not interact (i.e., independent) under the case-control status. Experiments on semi-empirical data showed that the distance correlation with permutation strategy yielded better power to detect underlying gene-based gene–gene interactions in a large range of settings, and the application to real datasets verified that GBDcor identified gene–gene interactions accurately.

## 2. Materials and Methods

In this section, we detail the statistical procedure for GBDcor. We then present the various settings for semi-empirical simulation studies for the type-I error rate and for the power to detect gene–gene interaction. Finally, we describe a real rheumatoid arthritis dataset from the wellcome trust case control consortium (WTCCC) database to evaluate our method in a real situation.

### 2.1. GBDcor

#### 2.1.1. Preliminaries and Notation

Suppose that we have random samples:

$$\left(G_{1,i},G_{2,i}\right)\in R^{p+q},i=1,2,...,n$$

where:

$$G_{1,i}=\left(g_{1,i,1},g_{1,i,2},...,g_{1,i,p}\right),G_{2,i}=\left(g_{2,i,1},g_{2,i,2},...,g_{2,i,q}\right),i=1,2,...,n$$

where $G_{1}$ and $G_{2}$ represent genes with p and q SNPs, respectively. $g_{k,i,j}\in \left\{0,1,2\right\}$ is the number of copies of the minor allele for SNP *j* in gene *k* of sample *i*. We focus on the case-control data that $y_{i}\in \left\{0,1\right\}$ is a categorical label, where 1 represents case subjects and 0 represents control subjects. Here, $G_{1}$ and $G_{2}$ are assumed to take values in ${\left\{0,1,2\right\}}^{p}$ and ${\left\{0,1,2\right\}}^{q}$, respectively, where $\left(G_{1,i},G_{2,i}\right)\in {\left\{0,1,2\right\}}^{p+q},i=1,2,...,n$, is a random sample from the joint distribution of $\left(G_{1},G_{2}\right)$.

In this work, to investigate whether there is a statistical interaction between two genes in a qualitative phenotype, we combine the distance correlation with the permutation strategy to test whether two genes interact. Our approach is based on the intuition that, if there is no interaction between two genes, then, if they are independent of the case set, then they should be independent of the control set; if they are dependent on the case set, they should be dependent on the control set also, and the “strength” of such dependence should be the same on the case and control set. The degree of dependence between two random variables can be measured by Pearson’s correlation coefficients. However, it can only measure linear dependency and not nonlinear dependency, and it is not very convenient for random variables that take a value in $R^{n}$; hence, we propose measuring them by the distance correlation coefficient instead.

#### 2.1.2. Distance Correlation

Let X and Y be two random variables in $R^{n}$ with finite first moments, then their distance covariance, denoted by dCov(X,Y), and distance correlation coefficients, denoted by $R^{2}\left(X,Y\right)$, are defined in ([33]). They satisfy the following properties:

- R(X,Y) is defined for X and Y in arbitrary dimensions.
- $R^{2}\left(X,Y\right)=\frac{dCov^{2}\left(X,Y\right)}{\sqrt{dCov^{2}\left(X,X\right)dCov^{2}\left(Y,Y\right)}}$
- R(X,Y)=0 if and only if X and Y are independent.
- 0≤R(X,Y)≤1

The proofs can be found in [33]. In particular, Property 4 above tells us that the distance correlation can be used to measure the degree of dependency between two random variables.

If there are *n* samples $(X_{i},Y_{i}),i=1,...,n$, according to [33], the distance covariance and distance correlation can be estimated by the sample distance covariance and sample distance correlation, which we will describe below.

Let $A_{i,j},B_{i,j}$ be the centered distance matrix of the samples $X_{i},Y_{i}$. In other words,

(1) $$A_{i,j}=|X_{i}-X_{j}|-\frac{1}{n}\sum_{k}|X_{k}-X_{j}|-\frac{1}{n}\sum_{l}|X_{i}-X_{l}|+\frac{1}{n^{2}}\sum_{k,l}\left|X_{k}-X_{l}\right|$$

(2) $$B_{i,j}=|Y_{i}-Y_{j}|-\frac{1}{n}\sum_{k}|Y_{k}-Y_{j}|-\frac{1}{n}\sum_{l}|Y_{i}-Y_{l}|+\frac{1}{n^{2}}\sum_{k,l}\left|Y_{k}-Y_{l}\right|$$

Then, the sample distance covariance is defined as:

(3) $$dCov_{n}^{2}\left(X,Y\right)=\frac{1}{n^{2}}\sum_{i,j}A_{i,j}B_{i,j}$$

and the sample distance correlation coefficient is:

(4) $$R_{n}^{2}\left(X,Y\right)=\frac{dCov_{n}^{2}\left(X,Y\right)}{\sqrt{dCov_{n}^{2}\left(X,X\right)dCov_{n}^{2}\left(Y,Y\right)}}$$

#### 2.1.3. dCor with Permutation Strategy

Assume there are $n_{1}$ cases and $n_{2}$ controls in a case-control study for a pair of genes $G_{1}$ with p SNPs and $G_{2}$ with q SNPs. Let $dCor_{n}=R_{n}^{2}\left(G_{1},G_{2}\right)$ be the sample distance correlation between Gene 1 and Gene 2 for a subsample of size *n*. First, we calculate the $dCor_{n_{1}}^{C}=R_{n_{1}}^{2}\left(G_{1}^{C},G_{2}^{C}\right)$, $dCor_{n_{2}}^{D}=R_{n_{2}}^{2}\left(G_{1}^{D},G_{2}^{D}\right)$. Second, we design a statistic $\Delta dCor=\frac{|dCor_{n_{1}}^{C}-dCor_{n_{2}}^{D}|}{dCor_{n_{2}}^{D}}$ to measure the difference in distance correlations between cases and controls. This represents how different the two joint distributions $\left(G_{1}^{C},G_{2}^{C}\right)$ and $\left(G_{1}^{D},G_{2}^{D}\right)$ are. The larger the ΔdCor, the higher the probability that Gene 1 and Gene 2 interact.

Because we have no information about the distribution of our designed statistic, it is difficult to use a conventional parametric test to do the statistical inference. Therefore, we apply the permutation strategy to estimate the significance of gene–gene interaction. During the permutation test, we rearrange label y to generate a new random case and control label, calculate ΔdCor, construct the empirical distribution, and estimate the *p*-value. We do the permutation *m* times and get $\Delta dCor^{1},\Delta dCor^{2},...,\Delta dCor^{m}$. The statistic for the original dataset is $\Delta dCor^{0}$

Here, the null hypothesis and the alternative hypothesis are defined as follows:

$$H_{0}:\Delta dCor^{i}\;\mathrm{has}\;\mathrm{the}\;\mathrm{same}\;\mathrm{distribution}$$

(5) $$H_{1}:\Delta dCor^{0}\;\mathrm{has}\;a\;\mathrm{distribution}\;\mathrm{different}\;\mathrm{from}\;\mathrm{the}\;\mathrm{other}\;\Delta dCor^{i}$$

After the permutation, the random samples follow the null hypothesis $H_{0}$. According to *m* statistics from random permutation samples, we can derive the sampling distribution (i.e., empirical distribution) for the statistic ΔdCor following the null hypothesis $H_{0}$.

We count the number of statistics $\Delta dCor^{i}$ that are equal to or greater than $\Delta dCor^{0}$.

$$num=\sum_{i=1}^{m}I\left(\Delta dCor^{i}\geq \Delta dCor^{0}\right)$$

(6) $$I\left(\Delta dCor^{i}\geq \Delta dCor^{0}\right)=\left\{\begin{array}{c} 1,\Delta dCor^{i}\geq \Delta dCor^{0}\\ 0,\Delta dCor^{i}<\Delta dCor^{0} \end{array}\right.$$

Then, we estimate the *p*-value by:

(7) $$p=\frac{num}{m}$$

The framework for GBDcor is described in Algorithm 1. 

**Algorithm 1:** GBDcor

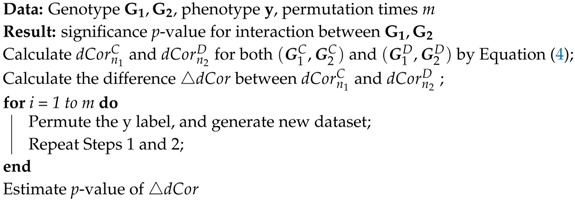



### 2.2. Simulation Study

To evaluate the power to detect gene–gene interaction and the ability to control the type-I error rate of GBDcor, we compared GBDcor with three existing techniques: kernel canonical correlation analysis (KCCA) [28,29], the gene-based information gain method (GBIGM) [30], and gene-based gene-gene interaction test (AGGrEGATOr) [31].

#### 2.2.1. Simulation Based on Haplotype Population

**Figure 1:**
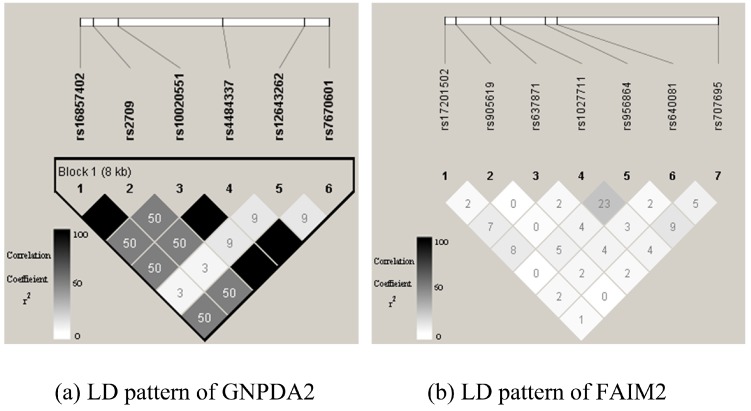
Linkage disequilibrium (LD) patterns of GNPDA2 and FAIM2 used in simulation studies. Figures are LD plots produced using Haploview. GNPDA2 has 6 SNPs, and FAIM2 has 7 SNPs. The number in each square is the LD strength that was measured by $r^{2}$, where $0\leq r^{2}\leq 1$, 0 means no LD, and 1 means complete LD.

Here, we used gs2.0 to generate the simulation data. gs2.0 [38] takes haplotypes as input, then generates dense SNP genotype data for case-control samples that share similar local linkage disequilibrium (LD) patterns as those in human populations. By varying the odds ratio (OR), population prevalence, and sample size, it can generate different disease models. To mimic the real LD structure in a human population, we selected the U.S. Utah residents with ancestry from Northern and Western Europe from the CEPH collection (CEU population) of Hapmap3 (https://www.sanger.ac.uk/resources/downloads/human/hapmap3.html) as template haplotype data. The CEU dataset contains 90 haplotypes. In this study, we chose two gene loci randomly, GNPDA2 on chromosome 4 and FAIM2 on chromosome 12. We imputed chromosome 4 and chromosome 12 using the genipe module, which is a genome-wide imputation pipeline that uses Plink, shapeit, and impute2, with the 1000 Genome Project phase3 data as reference data. After imputation, we got 6 SNPs in GNPDA2 and 7 SNPs in FAIM2 (Table 1 Figure 1).

#### 2.2.2. Disease Model

A disease model is a model that expresses the relationship between genes and the disease. Here, we considered two-locus disease models. We take a jointly recessive-recessive model as an example. Suppose population prevalence is p and the genotype odds ratio is (1+θ) for each locus (Table 2).

Let $Pr(D|g_{i})$ denote the probability of a sample being affected given the genotype $g_{i}$ (i.e., the penetrance of $g_{i}$), and let $Pr(\bar{D}|g_{i})$ denote the probability of a sample not being affected given genotype $g_{i}$. Therefore, the odds of a disease can be written as follows:

(8) $$ODD_{g_{i}}=\frac{Pr(D|g_{i})}{Pr(\bar{D}|g_{i})}=\frac{Pr(D|g_{i})}{1-Pr(D|g_{i})}$$

The penetrance of genotype $g_{i}$ can be calculated:

(9) $$Pr\left(g_{i}\right)=\frac{ODD_{g_{i}}}{1+ODD_{g_{i}}}$$

Table 3 is the corresponding penetrance table for Table 2.

Once the population prevalence p and the genotype odds ratio (1+θ) are fixed in this model, we can calculate the baseline value γ, which represents the odds of disease when the two loci do not carry any disease alleles, by using the following formula and the terms in Table 3.

(10) $$p=Pr\left(D\right)=\sum Pr\left(D|g_{i}\right)\times Pr\left(g_{i}\right)$$

We used eight build-in disease models in gs2.0, which included an additive-additive model, recessive-recessive model, threshold model, XOR model, dominant-dominant model, multiplicative model, recessive-dominant model, and a special interaction model. By varying population prevalence, odds ratio, and sample size, we generated different datasets to perform a comparative analysis of AGGrEGATOr, KCCU, and GBIGM.

Type-I error: Type-I error is the probability of rejecting the null hypothesis when the null hypothesis is true. In this paper, we set the significance level at α=0.05. We performed the simulation 100 times with each sample size n∈{1k,2k,3k,4k,5k}, by setting the odds ratio at 1.

Power: The power of a statistical test is the probability that it rejects the null hypothesis correctly when the alternative hypothesis is true. In this paper, we ran the simulations 100 times for each parameter combination. The power for each parameter combination is the frequency of rejection of the null hypothesis in the dataset when the alternative hypothesis is true under the significance level of α=0.05. To evaluate the effect of the odds ratio, we varied the odds ratio OR∈{1.5,2,2.5,3,3.5,4} with population prevalence at p=0.01 and a sample size of k=4000 (2000 cases and 2000 controls). To evaluate the effect of sample size, we choose n∈{1k,2k,3k,4k,5k} with an odds ratio of OR=2 and population prevalence of p=0.01.

For GBDcor, AGGrEGATOr, KCCU, and GBIGM, if the number of datasets with a *p*-value less than α was $m_{1}$, the power was calculated by:

(11) $$power=\frac{m_{1}}{100}$$

For GBDcor, AGGrEGATOr, and GBIGM, we used a nonparametric method with no parameter specified. For KCCU, we set the ratio for a trimmed jackknife at 0.05 (ω=0.05).

### 2.3. Application with Rheumatoid Arthritis Data

To assess the capacity of GBDcor to deal with real gene–gene interaction of a case-control dataset, we investigated the susceptibility of a set of pair of genes in rheumatoid arthritis (RA), which is a chronic, autoimmune joint disease where persistent inflammation affects bone remodeling and results in progressive bone destruction. We used the WTCCC (2007) dataset, which was genotyped in a British population using the Affymetrix GeneChip 500k.

To verify our method, we constructed our dataset in the following ways:

(1) We wanted to verify some gene–gene interaction in the RA pathway hsa05323 in the KEGG pathway dataset. Genotyping coordinates are given in NCBI Build36/UCSC hg18 (National Center for Biotechnology Information, Bethesda, MD, USA). There is a total of 90 genes in this pathway. Because MHCII and V-ATPase are two protein combinations with many interactions within themselves, we only chose a representative gene from each of them and excluded other genes. After that, 48 genes were left. Each unique gene pair was evaluated, which resulted in a total of 482=1128 pairs for those genes.
(2) We obtained gene information from the NCBI Build36 annotation file. For each gene, 10 kb of buffer region were added both upstream and downstream of the defined gene location. All SNPs between the regions were considered.
(3) Based on the quality control provided by GWAS, we removed samples where the reported sex did not match the heterozygosity rates observed on chromosome X. We also excluded an SNP if its minor allele frequency (MAF) was <0.05, if its missing rate was >10% of the samples, or if its frequencies in the control were not in Hardy–Weinberg equilibrium (*p* < 0.0001). After filtering, there were 385 SNPs left in 4966 samples, which consisted of 1973 cases and 2993 controls.

## 3. Results

### 3.1. Simulation Study

#### 3.1.1. Type-I Error

After we set the significance level at α=0.05, changing the sample size gradually resulted in type-I errors for all the methods that were close to the significance level for most sample size settings (Table 4), except for GBIGM at n=1k. The type-I error was controlled by these methods with different sample sizes with no effects.

#### 3.1.2. Power

**Figure 2:**
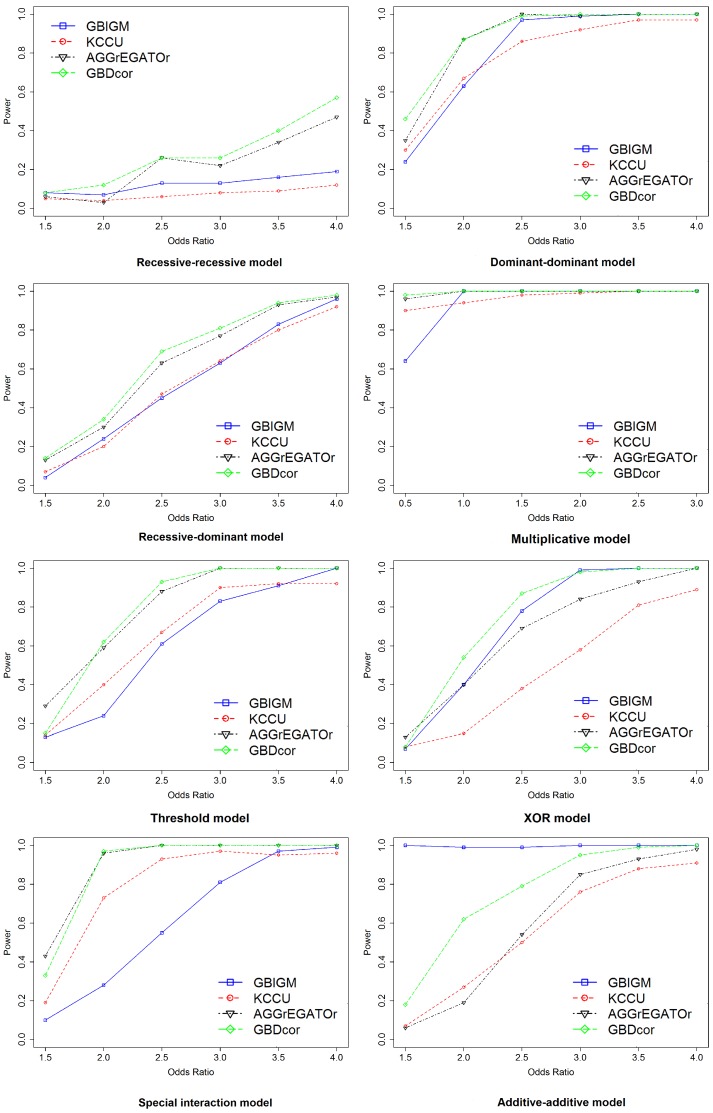
Empirical, simulation-based statistical power of GBIGM, KCCU, AGGrEGATOr, and GBDcor under eight disease models, after varying the OR∈{1.5,2,2.5,3,3.5,4}.

The effect of the odds ratio: We assessed the performance in detecting gene–gene interaction under eight disease models. The curves were constructed while varying the odds ratio (OR∈{1.5,2,2.5,3,3.5,4}) with population prevalence set at 0.01 and sample size set at 4k (Figure 2). Notice that a larger power indicated better performance. For this experiment, we chose one pair of SNPs belonging to different genes randomly to be causal to generate the simulated dataset. We considered the two genes that contain the SNPs to be interacting. The performance of all methods improved when OR became larger, and the power tended to be one for some methods at OR=4. Among them, GBDcor was the best, except for the additive-additive model (AA model). GBIGM showed the best performance under this model; however, it has been declared that GBIGM had a fatal inflation problem under this disease model. We also noticed that for the recessive-recessive model (RR model), when the OR value changed gradually from 1.5–4, the power was consistently ≤20%. AGGrEGATOr reached 40%, and GBDcor was approximately 60%. According to the penetrance table for the recessive-recessive model (Table 3), when we set population prevalence p=0.01, the baseline γ was a very small number. Therefore, among nine genotypes, eight of them tended to be zero. The only genotype (aabb) that was causal consisted of two minor alleles. Usually, the minor allele frequency of SNP was 0.2–0.4, which caused the genotype (aabb) to emerge only barely in the simulation dataset. Therefore, it was difficult to see any difference between cases and controls. That is, these methods showed poor performance under this model.

**Figure 3:**
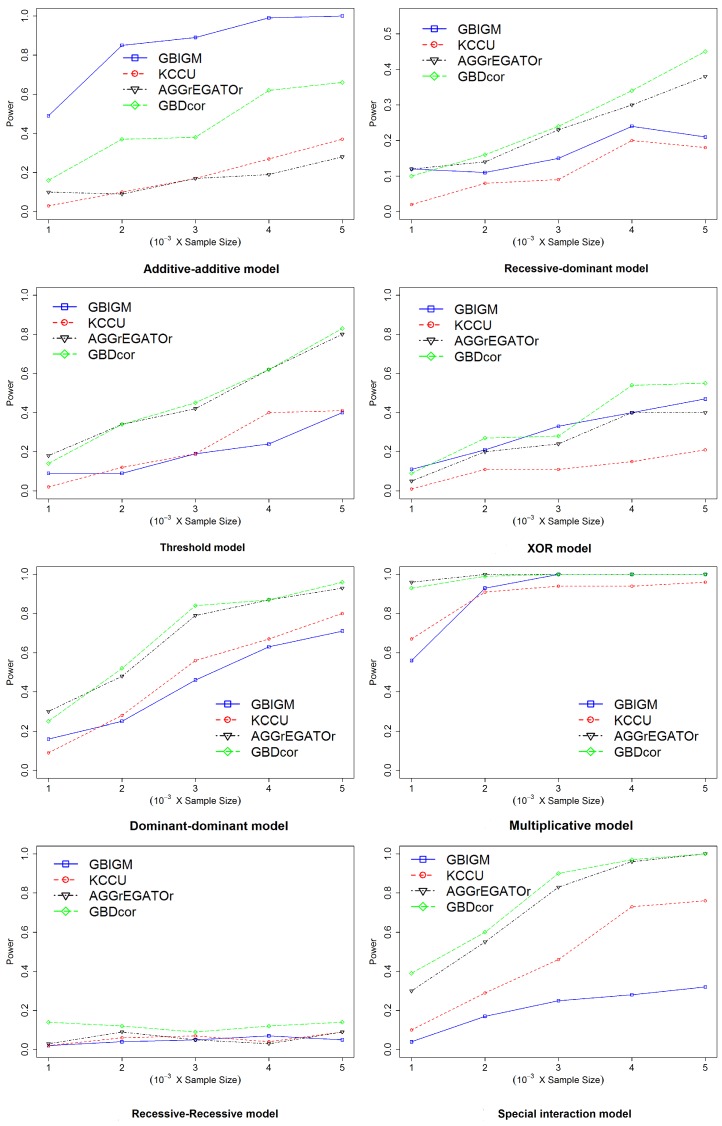
Empirical, simulation-based statistical power of GBIGM, KCCU, AGGrEGATOr, and GBDcor under eight disease models, after varying the n∈{1k,2k,3k,4k,5k}.

The effect of sample size: Next, we explored the impact of sample size. We set sample size n∈{1k,2k,3k,4k,5k} with OR=2 and p=0.01 (Figure 3). With increasing sample size, the power of all the methods increased monotonically under all disease models, except the RR model. Other than GBIGM, GBDcor performed much better than KCCU or AGGrEGATOr under the AA model. The power of GBDcor reached 60%, but the other two methods were ≤30%. For all methods, larger sample size led to better performance.

GBDcor performed better than alternative methods for almost the entire range of settings that we used. The benefits of using distance correlation to learn the dependence relationship of two genes in cases and controls were pronounced in the gene–gene interaction detection scenario. For example, we were able to design a statistic to represent the degree of difference of the two distance correlation coefficients and to apply a permutation to find the empirical distribution of our designed statistic.

### 3.2. Application Using Rheumatoid Arthritis Data

Rheumatoid arthritis (RA) is an autoimmune synovitis characterized by the formation of panus and destruction of cartilage and bone in synovial joints. TNF-α, IL-6, IL-17, MMPs, and RANK are some of the main players in the development of RA [39]. For the RA study of the hsa05323 pathway, we obtained 1128 pairs of genes to evaluate. For our method, we did permutation m=1000 times. Using a significance level of α=0.01, KCCU and GBIGM obtained 159 and 134 significant gene–gene interaction (GGIs), respectively. Thirty and 65 had a *p*-value equal to zero, respectively. AGGrEGATOr had 17 significant GGIs, and GBDcor had 18 significant GGIs.

Because GBIGM and KCCU had too many pairs, we were unable to analyze all of them. We selected the top 10 in GBDcor and in AGGrEGATOr for analysis. Then, we listed the *p*-value for each of the 20 pairs of genes for each of the methods (Table 5). We found seven of 10 results for GBDcor in the literature that supported our results, and we found two of 10 for AGGrEGATOr that did so. The column ‘Ref’ in Table 5 gives the references for the literature evidence that show direct interaction between two genes. We also observed that there were more intersections among GBDcor, KCCU, and GBIGM than among AGGrEGATOr, KCCU, and GBIGM.

## 4. Conclusions

Case-control datasets are common and important in research in medicine and evolutionary biology. In this paper, we developed a gene-based, gene–gene interaction detection algorithm called GBDcor that was based on distance correlation coefficients and a permutation strategy for GWAS on case-control datasets. The method benefits from the ability of distance correlation coefficients, which can detect nonlinear models, and the robustness of our hypothesis testing scheme, which is based on permutation and is non-parametric.

As a consequence, GBDcor was able to detect interpretable gene–gene interaction more accurately and effectively compared to other methods. We demonstrated such effectiveness through a semi-empirical simulation study and retrospective analysis of rheumatoid arthritis. Under a large range of settings, GBDcor outperformed previous methods in the power of detecting gene–gene interaction. The method was also stable to sample size based on a test of false positive rates. The distance correlation had no limitation on the dimension of two random vectors. Therefore, it is possible to generalize the method for pairwise, marker-based detection of gene pairs that were identified as interactive. Investigating the mechanism of gene-level interaction at the marker-level might be a direction for further research. In summary, GBDcor is a useful addition to the current toolbox of statistical models for unraveling gene–gene interaction in case-control studies.

## Figures and Tables

**Table 1:** Detailed information about GNPDA2 and FAIM2 used in a study of gene–gene interaction. Shown are the rsID (rs number used by researchers and databases to refer to specific SNPs)and physics position of each SNP on each gene.

Index	SNP Name: Position	
	GNPDA2 (chr4)	FAIM2 (chr12)
1	rs16857402:44706453	rs17201502:50285562
2	rs2709:44706913	rs905619:50286055
3	rs10020551:44707815	rs637871:50287592
4	rs4484337:44711547	rs1027711:50288032
5	rs12643262:44714455	rs956864:50290023
6	rs7670601:44715341	rs640081:50290554
7		rs707695:50297670

**Table 2:** Odds table of the recessive-recessive model.

SNP1		SNP2	
	BB	Bb	bb
AA	γ	γ	γ
Aa	γ	γ	γ
aa	γ	γ	γ(1+θ)

**Table 3:** Penetrance table of the recessive-recessive model.

SNP1		SNP2	
	BB	Bb	bb
AA	$\frac{\gamma }{1+\gamma }$	$\frac{\gamma }{1+\gamma }$	$\frac{\gamma }{1+\gamma }$
Aa	$\frac{\gamma }{1+\gamma }$	$\frac{\gamma }{1+\gamma }$	$\frac{\gamma }{1+\gamma }$
aa	$\frac{\gamma }{1+\gamma }$	$\frac{\gamma }{1+\gamma }$	$\frac{\gamma (1+\theta )}{1+\gamma (1+\theta )}$

**Table 4:** Type-I error of the four methods in different sample sizes. AGGrEGATOr, a gene-based gene gene interaction; GBDcor, gene-based gene-gene interaction via distance correlation coefficient GBIGM, gene-based information gain method; KCCU, kernelized CCU.

Method	Sample Size				
	1*k*	2*k*	3*k*	4*k*	5*k*
AGGrEGATOr	0.05	0.06	0.07	0.04	0.02
GBDcor	0.05	0.03	0.04	0.04	0.06
GBIGM	0.13	0.06	0.07	0.07	0.07
KCCU	0.02	0.02	0.01	0.05	0.07

**Table 5:** The *p*-values of the gene pairs detected to interact from different methods. The *p*-values with bold font mean they are significant

Gene1	Gene2	Ref	*p*-Value			
			GBDcor	AGGrEGATOr	KCCU	GBIGM
AP-1	M-CSF	ref [40]	**0**	0.0679	**0.001**	**0**
CXCL12	FLT-1		**0**	0.59	0.152	**0**
GM-CSF	VEGF	ref [41]	**0.001**	0.284	**0.005**	0.545
CTSK	VEGF		**0.002**	0.873	**0.028**	0.47
CTLA4	TLR2		**0.002**	0.152	0.057	**0.008**
CXCL1	RANK	ref [42,43,44]	**0.002**	**0.024**	0.147	0.697
IL15	MMP-3	ref [45]	**0.002**	0.066	0.167	0.088
GM-CSF	AP-1	ref [46,47]	**0.002**	0.394	**0.001**	**0.027**
CD86	APRIL	ref [48]	**0.003**	0.637	**0.03**	0.655
TGFβ	VEGF	ref [40]	**0.005**	1	**0.029**	0.632
CD80	APRIL	ref [48]	0.865	**0.0006**	0.941	0.334
CTSK	BLyS		0.298	**0.0008**	0.356	0.056
AP-1	IL-6		0.24	**0.0018**	0.098	0.287
CD80	CTSL		0.094	**0.0019**	0.519	0.252
CXCL6	FLT-1		0.441	**0.0023**	**0.004**	0.52
CTLA4	AP-1		0.075	**0.0023**	**0.042**	0.102
FLT1	LFA-1		0.645	**0.0031**	0.063	**0.028**
CCL3	TRAP		0.746	**0.0032**	0.682	**0**
IL-18	TGFβ		0.841	**0.0036**	0.149	0.22
IL-1	SDF-1	ref [49]	0.618	**0.004**	0.116	0.636
